# Pre-amplification in the context of high-throughput qPCR gene expression experiment

**DOI:** 10.1186/s12867-015-0033-9

**Published:** 2015-03-11

**Authors:** Vlasta Korenková, Justin Scott, Vendula Novosadová, Marie Jindřichová, Lucie Langerová, David Švec, Monika Šídová, Robert Sjöback

**Affiliations:** Laboratory of Gene Expression, Institute of Biotechnology, Academy of Sciences of the Czech Republic, Prague, Czech Republic; QFAB Bioinformatics, University of Queensland - St Lucia QLD, Brisbane, Australia; TATAA Biocenter, Göthenburg, Sweden

**Keywords:** High-throughput qPCR, Exponential pre-amplification, Microfluidics, Gene expression, Fluidigm, BioMark, Degraded samples, FFPE

## Abstract

**Background:**

With the introduction of the first high-throughput qPCR instrument on the market it became possible to perform thousands of reactions in a single run compared to the previous hundreds. In the high-throughput reaction, only limited volumes of highly concentrated cDNA or DNA samples can be added. This necessity can be solved by pre-amplification, which became a part of the high-throughput experimental workflow. Here, we focused our attention on the limits of the specific target pre-amplification reaction and propose the optimal, general setup for gene expression experiment using BioMark instrument (Fluidigm).

**Results:**

For evaluating different pre-amplification factors following conditions were combined: four human blood samples from healthy donors and five transcripts having high to low expression levels; each cDNA sample was pre-amplified at four cycles (15, 18, 21, and 24) and five concentrations (equivalent to 0.078 ng, 0.32 ng, 1.25 ng, 5 ng, and 20 ng of total RNA). Factors identified as critical for a success of cDNA pre-amplification were cycle of pre-amplification, total RNA concentration, and type of gene. The selected pre-amplification reactions were further tested for optimal Cq distribution in a BioMark Array. The following concentrations combined with pre-amplification cycles were optimal for good quality samples: 20 ng of total RNA with 15 cycles of pre-amplification, 20x and 40x diluted; and 5 ng and 20 ng of total RNA with 18 cycles of pre-amplification, both 20x and 40x diluted.

**Conclusions:**

We set up upper limits for the bulk gene expression experiment using gene expression Dynamic Array and provided an easy-to-obtain tool for measuring of pre-amplification success. We also showed that variability of the pre-amplification, introduced into the experimental workflow of reverse transcription-qPCR, is lower than variability caused by the reverse transcription step.

**Electronic supplementary material:**

The online version of this article (doi:10.1186/s12867-015-0033-9) contains supplementary material, which is available to authorized users.

## Background

The popularity of real time PCR steadily increases as well as the number of platforms, detection chemistries and multiple choices of analytical methods. Several years ago, the boom in high-throughput instruments changed the way of studying gene expression and enabled researchers to perform large scale studies based on the most sensitive and specific quantitative PCR method. The first commercially available high-throughput qPCR instrument was the BioMark™ System from Fluidigm that was launched in 2006. Microfluidic Dynamic Arrays provided by Fluidigm are able to combine either 48 samples with 48 assays or 96 samples with 96 assays in a combinatorial manner inside the integrated fluidic circuit (IFC) [1]. The BioMark System is able to process a high number of reactions (9,216) in a single run, each reaction taking place in volume of 6.7 nl [2]. With this number of reactions in a single run and its versatility and the freedom of the custom designed assays, BioMark System outperforms other high-throughput qPCR systems. There are only a few high-throughput qPCR instruments on the market that can be compared with BioMark System: OpenArray using a chip with 3,072 reactions, each for 33-nanolitre reaction volumes (Life Technologies) [3] and SmartChip with 5,184 reactions, each for 100-nanolitre reaction volumes (Wafergen) [4]. All these systems are designed to significantly simplify experimental workflow, increase throughput and reduce costs, while providing excellent data quality. Even though these instruments are built on different platforms, one attribute is common for all of them and that is a need for highly concentrated starting sample material.

The problem with an insufficient number of copies of the target in the reaction can be overcome with the help of pre-amplification. For the purposes of BioMark System a specific target amplification (also known as STA) is used, which is a multiplex PCR run with cDNA template and with a limited number of cycles, which is an exponential type of pre-amplification enabling simultaneous gene expression measuring of multiple targets in a single sample [5-7]. This kind of pre-amplification increases the amount of the initial cDNA or DNA template molecules several-fold, quantitatively amplifies just the target genes to be measured, and preserves the relationships between the transcripts. Even though pre-amplification has been used for many years [8,9] and it has been incorporated in high-throughput qPCR instruments workflows [10-13], it is still the least studied part of qPCR workflow that might introduce an additional bias if it is used without caution and appropriate controls.

In last few years, we witnessed that along with new techniques and new bioinformatic approaches come praiseworthy effort for proper standardization and control of the whole experimental process to eliminate widespread publication of poor data, resulting in inappropriate conclusions [14]. Because of the initiator of the whole process, MIQE guidelines [15], the quality and transparency of the laboratory results has been improved considerably. Pre-amplification process should not be omitted from this effort and it should be thoroughly validated and correctly reported as well as other parts of reverse transcription-qPCR workflow. It means that controls of pre-amplification should include at least paired non-preamplified and pre-amplified samples and each assay should be tested independently before the main experiment as described by Rusnakova [16]. For unbiased pre-amplification, the same difference between Cq values of non-preamplified and pre-amplified cDNA samples is expected for all assays; only reproducible small deviations are acceptable. Reproducibility is critical. Other controls as pre-amplified no template control (NTC) and pre-amplified control of reverse transcription without reverse transcriptase (RT-) should also be included. The reason is to ensure that quantification will not be influenced by eventual primer-dimer formation or by assays that would amplify gDNA. RT- control could be successfully replaced by a valid prime assay, which accurately corrects all reactions in BioMark Array for signals derived from gDNA using only one extra valid prime assay and pre-amplified genomic DNA (gDNA) [17]. As the pre-amplification reaction is a highly complex multiplex system (it is possible to pre-amplify almost limitless number of measured genes), simultaneous amplifications of the large number of targets may interfere; therefore it is necessary to use highly optimized qPCR assays with high efficiency and high precision and to run only a limited number of cycles and avoiding high-abundant targets if possible [18]. Even though it is possible to use fewer cycles of pre-amplification (10–14) for qPCR experiments with conventional qPCR instruments, high-throughput qPCR experiments require more than 14 pre-amplification cycles. Fluidigm advanced protocols recommend 14 cycles for conventional profiling [19] and 18 cycles for single-cell profiling [20]. These numbers of pre-amplification cycles are calculated for highly optimized assays but in practice pre-amplification PCR efficiencies are not close to 100% that is why these numbers are minimal and often suboptimal [18].

Here, we focus on identifying factors which influence the pre-amplification reaction and the pre-amplification limits, especially a limiting higher number of cycles for pre-amplification, which has not been studied systematically yet. Our aim is to find out the optimal conditions for BioMark Array that would give us an optimal distribution of quantifiable Cq values across the Array by using the proper amount of mRNA transferred into a reverse transcription reaction; the proper fraction of the cDNA used for pre-amplification and the proper fraction of the pre-amplified and correctly diluted cDNA, transferred into each sample well in BioMark Array.

## Results and discussion

### Evaluating variables in pre-amplification reaction using regular qPCR instrument

The primary purpose of pre-amplification is to enhance amount of input material, which can be, in some instances, very low even for conventional qPCR: single cell analysis [16,21], microRNA analysis [22], analysis of formalin-fixed, paraffine embedded tissues [23] or to enhance initial amount of material to be sufficient for high-throughput instrument [1]. The amount of pre-amplified transcripts correlates with the initial cDNA target copy numbers as has been shown previously for both good quality samples [24] and bad quality samples, e.g. formalin-fixed paraffin-embedded samples [23]. The exponential pre-amplification should not be affected by the quality of original RNA because the product of reverse transcription, cDNA molecule, is pre-amplified. That is why the quality of RNA will influence only the reverse transcription step.

Even though the pre-amplification reaction itself is quite simple, there are several factors that can influence the final result. To identify and evaluate these factors we performed pre-amplification experiment combining different conditions. We evaluated four donors and five genes having high, medium and low expression levels. The genes were FKBP, STK10, EIF3M, CD83, and RND1 and were selected as representative from 24 well-characterized assays (Additional file 1) that were used later on for the summarizing BioMark experiment. Their mean Cq values for four non-pre-amplified samples were 18.7, 21.5, 23.7, 26.7, 34.0, respectively, which expression is spanning four orders of magnitude of dynamic range. Each sample was pre-amplified using four different cycles (15, 18, 21, and 24) and at five different concentrations (equivalent to 0.078 ng, 0.32 ng, 1.25 ng, 5 ng, and 20 ng of total RNA). The copy number of each transcript and sample was estimated for each assay. The estimated copy number for the low expressed gene RND1 was confirmed by dPCR. The limit of detection (LOD), the limit of quantification (LOQ) and the efficiency were determined for all 5 assays (Additional file 1).

Obtained non-pre-amplified Cq data and pre-amplified Cq data were subtracted to calculate an ‘experimental difference’ of pre-amplification: ΔCq_experimental_ = Cq_non-preamp_ – Cq_preamp_. A ‘theoretical difference’ of pre-amplification was calculated as: ΔCq_theoretical_ = number of pre-amplification cycles – log_2_ (all dilutions during the processing of the sample). The final formula was ΔΔCq = ΔCq_theoretical_ - ΔCq_experimental_. An obtained ΔΔCq value, ‘expression differential’, close to zero indicates pre-amplification uniformity (example of calculation in Additional file 2). We set ΔΔCq = 1.5 as a quality threshold for an acceptable pre-amplification. This threshold value is in agreement with the threshold value recommended by Applied Biosystems in TaqMan PreAmp Master Mix Kit guide [11]. The values lower than the quality threshold (≤ ±1.5) were named a ‘success’. The values higher than a quality threshold and the missing values, caused by missing copies in the reaction, were categorized as a ‘failure’ (16 or 4% of cases) (Additional file 2).

In order to evaluate which factors affect the ‘success’ of pre-amplification, we tested these data variables: Cycles (number of pre-amplification cycles), Log_copy (log_2_ copy number of cDNA used for pre-amplification), Log_concentn (log_2_ concentration of cDNA, presented as total RNA equivalent, used for pre-amplification), Donor, GeneNo (gene number = different transcripts) that were used in explanatory binomial candidate model. The optimal model was then derived in SPSS using the backward stepwise method to eliminate non-significant terms, which were Donor and Log_copy. Because all terms are known beforehand and controllable, the model could serve also as a predictive model with sensitivity of 81% and specifity 67% (Additional file 3).

Individual statistical tests uncovered important details of the pre-amplification process. Concentration of cDNA sample used for pre-amplification had significant effect on the overall likelihood of ‘success’ when tested for all Genes and Cycles together (p = 0.012); the higher Concentration, the higher ‘success’ (Additional file 4A). When individual Genes were taken in account and all Cycles were together, Concentration had significant effect only on low copy genes, RND1 (p < 0.001) and CD83 (p = 0.001) (Additional file 4B). Both genes show high failure rates in the low concentrated pre-amplification reactions (up to 5 ng) because the low template concentration corresponds to the low number of copies in pre-amplification (<10 copies of cDNA). These findings are in agreement with Bengtsson [25], who claims that when amplifying less than 20 cDNA copies the level of technical noise of PCR amplification increases dramatically, technical reproducibility decreases, thus the accurate quantification is reached if >20 target molecules per PCR are amplified.

Copy number of cDNA used for pre-amplification was not significant in the predictive model because Copy number (Log_copy) did not have a significant effect on the overall likelihood of ‘success’ when all Genes and all Cycles were combined together (p = 0.322) (Additional file 5A). However, if each Gene was tested independently with all Cycles together, the same results as for variable Concentration were obtained. Copy number had significant effect on low copy genes RND1 (p = 0.0001) and CD83 (p = 0.0004) (Additional file 5B). Additional information was derived if Copy number was compared for all Genes and each Cycle independently. Whereas the likelihood of ‘success’ increased with increasing Copy number for cycles 15 (p = 0.0006) and 18 (p = 0.0002), it decreases for cycle 24 (p = 0.0007). The contradictory directions for individual Cycles can explain why there was no overall significant effect above (Additional file 5C). The increasing ‘success‘of pre-amplification with higher Copy numbers has been described before, for example, using different copy numbers of ERCC RNA-42 standard with 14 cycles of pre-amplification [26]. However, the effect of high copy number transcripts combined with higher pre-amplification cycles (>18 cycles) has not been systematically investigated for bulk experiments before.

**Figure 1:**
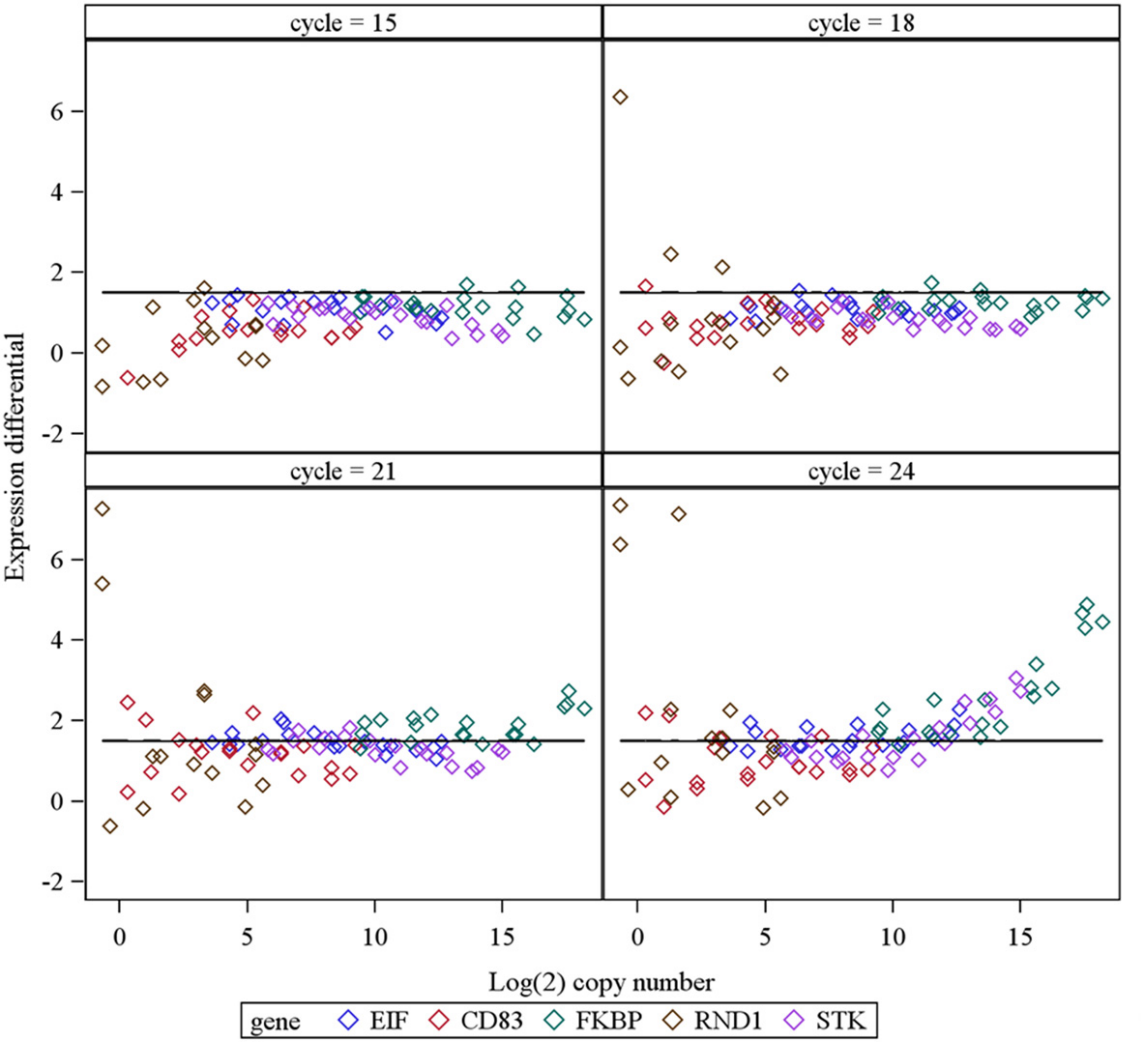
**A plot showing the quality of pre-amplification.** Successfully pre-amplified samples lie bellow the quality threshold, which corresponds to ΔΔCq = 1.5 (expression differential). The quality of pre-amplification gets worse with increasing number of pre-amplification cycles. During cycles 15 and 18 only small number of samples amplified with low copy gene RND1 (lowest concentrations) and high copy gene FKBP are affected. At cycles 21 and 24, the quality of pre-amplification is affected in all genes to some extent. The least affected gene is CD83, the most affected are high copy genes.

Finally, effect of number of Cycles on pre-amplification ‘success’ was tested. We show that the number of Cycles had a highly significant effect on overall likelihood of ‘success’ (p < 0.001) if tested for all Genes and Concentration together. Increasing Cycle numbers decreased the likelihood of ‘success’ (Additional file 6A). If both Genes and Cycles were tested independently, Cycle number had significant effect only on high copy genes EIF3M (p = 0.001), STK10 (p < 0.001) and FKBP (p < 0.001). Increasing Cycle numbers drastically decreased the likelihood of ‘success’ (Additional file 6B). The presence of highly abundant transcripts has also effect on pre-amplification process, this effect was combined with number of Cycles. While pre-amplifiying 21 and 24 cycles, the quality of pre-amplification steeply dropped, which is shown in summary figure (Figure 1). The percentage of affected genes displayed in this figure can be found in Additional file 7. This would probably be caused by getting under optimal concentration of primers in the multiplex pre-amplification reaction. The possibility of exhausting reagents during qPCR reaction was ruled out by testing limiting dilutions of PCR product of FKBP (data not shown).

Applying the results, we can speculate why 18 s rRNA, which is often used as a reference gene using conventional qPCR would not be suitable transcript for pre-amplification as was also suggested by Stahlberg [18]. The previously published data demonstrated that the highest correlation observed for samples pre-amplified with 18 s rRNA measured with microfluidic BioMark Array and non-preamplified samples measured by conventional qPCR cycler was 0.801 [27]. The expression of 18 s rRNA is so abundant that we recommend to exclude it from pre-amplification reaction completely. 18 s rRNA would not be detected reliably because of the very high concentration of transcripts present after pre-amplification. This reason would cause the inability of any instrument to set the correct baseline. On the other hand, for the same reason, it is possible to quantify 18 s rRNA in BioMark array without pre-amplification (Additional file 8). The simple clue for identifying possible unsuitable targets for pre-amplification is their measured Cq value. The Cq value of the non-preamplified high-abundant transcript should not be lower than the number of cycles being used for its pre-amplification.

After summarizing all results together, combination of significant variables Cycle and Concentration reveals that a cycle 15 or 18 combined with a concentration of 20 ng is the best pre-amplification option using good quality samples, although any concentration higher than 1.25 ng is likely to be sufficient if the cycle is 18 or 15 (Table 1). In other words, the solution is to minimize number of Cycles and maximize Concentration of the sample. Presented model (Table 1) can also be applied for degraded samples, e.g. formalin-fixed paraffin-embedded samples. If RNA samples are degraded, less cDNA could be formed during reverse transcription, thus less target copies of cDNA can be pre-amplified. Using our outcomes (recommended combinations of concentrations and cycles), the highly expressed transcripts will never be over-preamplificated. However, the right concentration and dilution of samples need to be tested for the low expressed genes.

**Table 1:** **A pivot table showing the success rate as a percentage for the possible combinations of Cycles (pre-amplification cycles) and concentrations for all five genes**

**Average of success**	**Concentration (equivalent of total RNA in ng used in pre-amplification)**					
**Cycles**	**0.078**	**0.32**	**1.25**	**5**	**20**	**Grand total**
15	75%	90%	90%	90%	100%	89%
18	80%	80%	90%	95%	100%	89%
21	40%	30%	60%	75%	80%	57%
24	45%	50%	55%	40%	40%	46%
Grand total	60%	63%	74%	75%	80%	70%

Cycle 18 combined with a Concentration of 5 and 20 ng, and Cycle 15 combined with a concentration of 20 ng is optimal for successful BioMark experiment.

### Optimal dilution for BioMark system

The regular BioMark high-throughput gene expression experiment consists of high number of assays (up to 48 or up to 96) [1] that are amplified at the same time, resulting in the big spread of Cq values from highly expressed to lowly expressed genes. If either the concentration of the sample, the number of pre-amplification cycles or dilution after pre-amplification are not set correctly, the final result will not be optimal. Some transcripts could be under-amplified, which can result in a loss of detection sensitivity and generation of missing values. We should also avoid over-cycling of highly expressed transcripts. If the concentration of copies for a certain assay is too high in the sample, the instrument might not be able to distinguish the background of the reaction and set the baseline properly. The obtained Cq values will not be reliable.

For a successful BioMark experiment, it is desirable for the majority of the data to fall in the range about Cq = 6 to Cq = 23 [28,29]. In contrast to the regular qPCR cyclers, the optimal range of quantifiable Cq values in BioMark instrument is approximately 10 cycles lower [26]. This is caused by the fundamental difference between the size of the surface that comes into contact with the sample mix during the qPCR reaction in the Dynamic Array and in the conventional micro-titer plate. In contrast to the polypropylene surface in conventional micro-titer plates, the percentage of surface of polydimethylsiloxane nanochamber [30] that is connected with the reaction volume is much larger. It leads to a higher sensitivity of the microfluidic system, earlier detection of the fluorescence signal and thus shorter cycling time.

**Figure 2:**
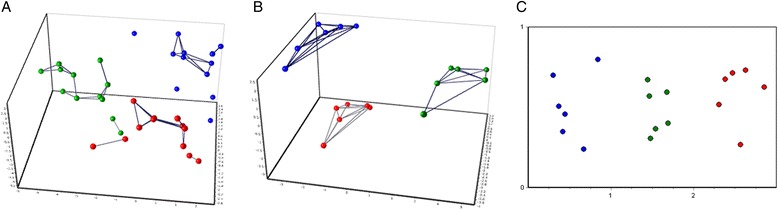
**Identification of the best concentration and dilution of pre-amplified samples for the BioMark experiment. A**. 3-D principal component analysis. PCA is based on 21 differentially expressed, auto-scaled genes, which classified pre-amplified samples into three groups according to donors (blue = donor 1, red = donor 2, green = donor 3). Samples pre-amplified 15, 18 and 21 cycles are shown, diluted both 20x and 40x. It is difficult to distinguish clearly 3 groups. **B**. Only acceptable pre-amplifications useful for BioMark GE Dynamic Array are selected: 15 cycles - 20 ng, dilution 20x and 40x, respectively; 18 cycles – 5 and 20 ng, dilution 20x and 40x, respectively. **C**. SOM with samples selected for Figure 2B, confirms 3 distinct groups.

In order to identify the best concentration and dilution of pre-amplified samples that would be suitable for the BioMark experiment, we performed an experiment with 48.48 GE Dynamic Array using already pre-amplified samples from previous experiment with cycles 15, 18 and 21 respectively and with the concentrations 1.25 ng, 5 ng and 20 ng, respectively. The samples were diluted 20 and 40 fold, respectively to determine the best conditions for BioMark instrument. All obtained Cq data (from 21 assays excluding 3 reference genes) was normalized with GAPDH, PPIB and GUSB reference genes, which should cancel out the differences among the different concentrations and different amplification cycles but not the natural variability among donors. We set these criteria: missing data were not acceptable, the lowest Cq in Dynamic Array should be 6 and three samples should be distinguished and fall into respective groups. That is why, the two lowest concentrations (1.25 ng and 5 ng) for 15 cycle pre-amplification were removed from the analysis immediately. These criteria helped us to set up the principal component analysis that was used to reduce the dimensionality of a data set, which consisted of the 21 normalized gene assays, 3 pre-amplification cycles, 2 dilutions and 3 concentrations of 3 samples. PCA data was auto-scaled to reduce the effect of variation in the overall expression levels of the different genes. Only samples that created three separated compact groups were selected for further analysis. After removal of all samples pre-amplified 21 cycles and samples pre-amplified 18 cycles of concentration 1.25 ng, the data set was reanalyzed and the first 3 components of PCA explained 78.4% variability of auto-scaled data set. The right choice of selected samples from PCA was validated with another method, Kohonen’s self-organizing feature map (SOM) that confirmed separation of samples into 3 distinct groups (Figure 2).

As a result, the highest concentration, 20 ng, for 15 cycles pre-amplification, both 20x and 40x diluted; and 5 ng and 20 ng concentrations for 18 cycles of pre-amplification, both 20x and 40x diluted fulfilled our criteria for successful and reliable pre-amplification and would give the best unbiased result with maximum detectable data for BioMark gene expression experiment.

### Pre-amplification variability

In order to demonstrate how the combination of optimal conditions for success of pre-amplification would affect variability, the yield and standard deviations (SD) of pre-amplified FKBP, STK10, EIF3M, CD83, and RND1 were measured by qPCR. Pre-amplification reactions were performed in replicates of four on one cDNA that was synthetized from the same RNA pool. SD_PRE_ of combined pre-amplification and qPCR (Table 2) was calculated as weighted sum of the SDs of the qPCR (SD_qPCR_) and SD of the pre-amplification reaction (SD_pre_). SD_PRE_ was in the range of 0.14 – 0.24, which corresponds to variability 10% - 17% in estimated number of cDNA molecules (averaged variability for all genes is 13%). Variability increases towards the low expressed genes with higher Cq values (Table 2), which is caused by well described statistical effects [31].

**Table 2:** **Comparison of reverse transcription (RT) and pre-amplification (PRE) variability**

	***RND1***	***CD83***	***EIF3M***	***STK10***	***FKBP***	**AVG var.**
Efficiency (E)	91%	100%	97%	94%	100%	
Cq RT	31.7	25.6	24.6	22.1	20.2	
$S{D}_{\mathrm{RT}}=\sqrt{SD{(rt)}^2+SD{(qPCR)}^2}$	0.38	0.41	0.24	0.40	0.29	
Variability RT = (1 + E)^SD^_RT_	28%	33%	18%	17%	22%	23.6%
Cq PRE*	23.6	17.1	16.2	13.4	12.1	
$S{D}_{PRE}=\sqrt{SD{(pre)}^2+SD{(qPCR)}^2}$	0.24	0.18	0.18	0.14	0.16	
Variability PRE = (1 + E)^SD^_PRE_	17%	13%	13%	10%	12%	13%
Expression Differential	1.2	0.7	1.0	0.7	1.3	

*Equivalent of 5 ng of total RNA was used in 18 cycle pre-amplification, pre-amplified cDNA diluted 40x.

Previously, it has been described that experimental variation in the reverse transcription-qPCR (without pre-amplification) is mainly attributable to the reverse transcription step [32]. To confirm this statement also for reverse transcription–qPCR with the additional pre-amplification step, we performed the experiment where the yield and standard deviations of cDNA synthesis of the FKBP, STK10, EIF3M, CD83, and RND1 were measured by qPCR. This time reverse transcription reactions were performed in replicates of four on material from the same RNA pool as in previous pre-amplification experiment (Table 2). SD_RT_ of combined reverse transcription and qPCR (Table 2) was calculated as weighted sum of the SDs of the qPCR (SD_qPCR_) and SD of the RT reaction (SD_rt_). SD_RT_ was in the range of 0.24 – 0.41, which corresponds to variability 17% - 33% in estimated number of cDNA molecules (averaged variability for all genes is 23.6%). After comparison, the pre-amplification variability within the reverse transcription-qPCR experiment is significantly lower (p = 0.015) than variability caused by cDNA synthesis step.

## Conclusion

In order to perform a valid experiment that would lead to reliable results, it is necessary to know both the capabilities and limitations of the used method and instrument. Even though BioMark instrument performs the regular qPCR reaction, we need to take some special properties into account when setting high-throughput qPCR experiment. The most distinct deviation from the regular qPCR experiment workflow is the necessity of pre-amplification.

As has been demonstrated, pre-amplification success is based on several variables, the most important ones are number of pre-amplification cycles, concentration of the sample used for pre-amplification, and the gene itself. After testing possible combinations of these variables, we came to the conclusion that pre-amplification for the BioMark System using good quality samples is optimal between 15–18 pre-amplification cycles and higher concentrations of cDNA samples (5–20 ng of transcribed total RNA per pre-amplification reaction) diluted either 20x or 40x after pre-amplification. Use of higher amplification cycles (21 or 24) in bulk experiments (not in single cell experiments) is very limited because high abundant targets will cause an exhaustion of primers and reagents from pre-amplification reaction, thus they will cause lowering of pre-amplification success.

The success of the pre-amplification can be tested by our improved, easy-to-obtain, universal formula called “expression differential”. The algorithm, which is presented here, evaluates the "expression differential" based on a ΔΔCq value obtained subtracting ΔCq experimental - ΔCq expected or "theoretical". Formula can be used universally, for pre-testing of the quality of pre-amplification assays in high-throughput gene expression experiment as well as in RT-qPCR experiments with FFPE-RNA.

And finally, we show that variability of the pre-amplification, introduced into the experimental workflow of reverse transcription-qPCR, is lower than variability caused by the reverse transcription step.

## Methods

### Sample collection and preparation

Blood was collected in BD K_2_EDTA tubes (BD, cat. no. 367525), 10 ml draw volume, from healthy volunteers. After approval by Norwegian south east regional committee for medical and health research ethics (REC South East), all participants signed a written informed consent before participating in the study in accordance with the Helsinki declaration. As soon as possible after the first blood tube collection, EDTA blood from each volunteer was transferred to and PAXgene® Blood RNA Tubes (PAXgene) (PreAnalytiX) to maintain gene expression, incubated at room temperature for 2 hours, and then stored at −80°C.

### Isolation of RNA, quality control and reverse transcription

RNA from blood collected in the PAXgene tubes was extracted according to the standard protocol: PAXgene Blood RNA Kit (Qiagen) and stored at −80°C.

RNA quantity and purity was measured using NanoDropTM 1000 Spectrophotometer (Thermo Scientific). OD_260/280_ ratios for all samples were between 1.8 and 2.0. RNA integrity number (RIN) was checked using capillary electrophoresis performed on Agilent Bioanalyzer 2100, with RNA 6000 Nano Assay (Agilent Technologies). Sample 1 RIN = 8, sample 2 RIN = 7.3, sample 3 RIN = 7.7, sample 4 RIN = 7.6. Pooled sample 5 for variability modeling had RIN = 7.5.

cDNA synthesis was performed using High Capacity cDNA Reverse Transcription Kit (Life Technology) according to the manufacturer’s protocol with random hexamers in the final volume of 50 μl containing 500 ng total RNA using a cycler C1000 (Bio-Rad). cDNA samples were stored at −20°C and diluted just before use. For dilution of samples GenElute-LPA (Sigma Aldrich) diluted in 1xTE according to the manufacturer instructions was used.

### Primer and probe design

qPCR assays and a RND1 probe were designed by TATAA Biocenter, Sweden (Additional file 1). To avoid the amplification of genomic DNA all assays were placed to span and/or have one primer covering an intron/exon boundary. Criteria for the assays were: good linearity (5 log dynamic range at LC480 error < 0.2), efficiency (≥80%, ≤105%), specificity (no amplification of gDNA or at least 5 cycle’s difference between target and genomic Cq-value) and clear NTCs. All assays were initially evaluated with SYBR green chemistry to test the primers. After approval of the primers a hydrolysis probe for RND1 was designed and evaluated in the same way as described for the primers. PCR products were analyzed for specificity (single product) on a pre-made 2.2% agarose gel (Flash Gel system, Lonza). All primer designs were performed with Primer BLAST [33] followed by probe design with Beacon Designer® (PREMIER Biosoft International). Primers and the probe were ordered from Eurofins. Primers were HPSF purified. Probe was labelled with FAM as reporter and BHQ1 as quencher and HPLC purified.

### Real time PCR, copy number estimation, efficiency and limit of detection

10 μl qPCR reactions using SYBR green were prepared from 5 μl 2x TATAA SYBR GrandMaster Mix (TATAA Biocenter), 0.4 μl primers (final concentration 400 nM), 2.6 μl MB water, 2 μl cDNA (or pre-amplified cDNA diluted 20x). The qPCR was run in CFX384 (Bio-Rad) using the standard program 95°C for 1 min followed by 40 cycles 95°C for 3 s, 60°C for 60 s, and 72°C for 10s plus melting curve. At least triplicate qPCR reactions were performed for each qPCR experiment. Cq data were obtained by regression using Bio-Rad CFX Manager Software 3.0 (Bio-Rad).

For determination of the number of copies, PCR products were purified using QIAquick PCR Purification Kit (Oiagen) according to the manufacturer instructions, concentration was measured using Quibit® 2.0 Fluorometer (Life Technologies) and number of copies were calculated. Standard curves using PCR product of known copy numbers were generated and the copy numbers of tested samples were interpolated. The cDNA RND1 copy number for four donors was confirmed also by dPCR using a probe.

Limit of detections (LOD) was determined from standard curves with 6 replicates for each dilution, 8 dilutions 1:3 for assays: EIF3M, CD83, FKBP, RND1, STK10. (Additional file 1). Dilutions were made with carrier TE-LPA (Sigma Aldrich). The efficiency of remaining assays was determined from the standard curves generated from PCR product diluted 1:10 000 in TE-LPA with 3 replicates and 5 dilution 1:9 (Additional file 1). All standard curve experiments were run in CFX384 (Bio-Rad) with TATAA SYBR GrandMaster Mix (TATAA Biocenter).

### Gene specific pre-amplification for experiments using intercalating dye

A single aliquot of each cDNA sample (diluted in carrier TE-LPA), equivalent to 20 ng RNA, 5 ng RNA, 1.25 ng RNA, 0.32 ng RNA, 0.078 ng RNA, respectively, was used for pre-amplification with TATAA PreAmp GrandMaster® mix (TATAA Biocenter) at either 15 cycles, 18 cycles, 21 cycles or 24 cycles, respectively. The total volume of pre-amplification was 10 μl for each sample. The reaction contained 5 μl of pre-amplification mastermix, 2 μl of cDNA, 1 μl of pooled primers with a final concentration of each primer of 25 nM and 2 μl of MB water. The cDNA samples were subjected to pre-amplification. The following temperature protocol was used: 95°C for 30 s, followed by 15, 18, 21, 24 cycles, respectively at 95°C for 15 s and 60°C for 4 min. 24 assays were pre-amplified as multiplex and only 5 selected assays (see above) were tested in the experiment. A list of 24 assays used for pre-amplification is described in Additional file 1. As a control, water (NTC) was included in the pre-amplification reaction. The pre-amplified cDNA was immediately used or placed in freezer at −20°C. The pre-amplified cDNA was diluted prior to use at either 20x or 40x with MB water.

### High-throughput real time PCR with Eva green

qPCR was performed using the high-throughput platform BioMark™ HD System and the 48.48 GE Dynamic Arrays (Fluidigm) in duplicates in assays. 5 μL of Fluidigm sample premix consisted of 1 μL of either 20x or 40x diluted pre-amplified cDNA, 0.25 μL of 20x SG loading reagent (Fluidigm), 2.5 μL of Sso Fast Eva green mastermix (Bio-Rad), 0.1 μL of 4x diluted ROX (Invitrogen) and 1.15 μL of RNase/DNase-free water. Each 5 μL assay premix consisted of 2 μL of 10 μM primers (final concentration 400 nM primers), 2.5 μL 2x Assay loading reagent (Fluidigm) and 0.5 μL of RNase/DNase-free water. The samples and assays were mixed inside the chip using Nanoflex IFC controller (Fluidigm). Thermal conditions for qPCR were: 98°C for 40 s, 35 cycles of 95°C for 10 s, and 60°C for 40 s plus melting curve analysis. Data was processed by automatic threshold for each assay, with derivative baseline correction using BioMark Real-Time PCR Analysis Software 3.1.2 (Fluidigm). The quality threshold was set at the default setting of 0.65.

### qPCR data pre-processing and statistical analysis

The Cq data obtained from conventional qPCR cycler CFX384 was analyzed using IBM SPSS Statistics (Version 21) and an Excel (Version 14.3.4) pivot table. Tested variables were: Cycles (number of pre-amplification cycles), Log_copy (log_2_ copy number of cDNA used for pre-amplification), Log_concentn (log_2_ concentration of cDNA, presented as total RNA equivalent, used for pre-amplification), Donor, GeneNo (gene number = different transcripts). Copy number was analyzed as both a categorical and continuous variable. As the distribution of Copy number and Concentration were not normally distributed these were also log transformed (base 2). Each experiment was classified as a’success’ or’failure’. An experiment was classified as a’success’ if the’expression differential’ was less than ± 1.5 (Additional file 2). An experiment was classified as a’failure’ if the’expression differential’ was greater than 1.5 or missing.’Expression differential’ consisted of the’theoretical expression’ minus the’experimental expression’ (detailed description in results).

Measures of experiment behavior and outcome were compared against the likelihood of success to detect any statistical relationships. Univariate categorical measures were compared against experimental ‘success’ under specified conditions using the Chi-squared test (expected values were so high that the Fisher’s Exact test was not used). Univariate continuous measures were compared against experimental ‘success’ using Box plots and group summary tables. A full variable logistic regression model was pared back to an optimal model using the backward stepwise method by eliminating non-significant terms. A classification (or confusion) table was produced and the sensitivity and specificity calculated. A pivot table was produced showing the success rate as a percentage for the possible combinations of Cycles and Concentrations.

All BioMark data were pre-processed in the software GenEx Enterprise 5.4.0.520 (MultiD Analyses AB). PPIB, GAPDH and GUSB were selected for normalization using Normfinder software. Principal component analysis (PCA) [34] and Kohonen self-organizing maps (SOM) [35] was performed using 21 original independent variables (21 normalized genes). PCA and SOM were performed with data that were normalized, the lowest expression was recalculated to 1, log2 transformed and auto-scaled using GenEx Enterprise software. All expression values were auto-scaled in order to remove the influence of both the expression level and the magnitudes of the changes and gave rise to classification based on the relative changes in expression. The SOM of size 3 x 1 dividing the samples into 3 groups was trained using GenEx with the following parameters: 0.1 learning rate, 3 neighbors and 5,000 iterations. The SOM analysis was repeated five times with identical classification.

Difference between variability of reverse transcription step and pre-amplification step was tested by paired, two tailed *t* test using GenEx Enterprise 5.4.0.520 (MultiD Analyses AB).

## Additional files

Additional file 1:
**The Excel sheet with information on 24 assays used for pre-amplification.** Five assays highlighted in gray were used for experiment 3.1. Additional information are added for these assays: LOD, LOQ. Additional file 2:
**Table of all samples and their Cq and calculated characteristics as Concentration, Copy numbers, Cycle, Success and the example of the pre-amplification algorithm (expression differential) application.**
Additional file 3:
**Construction and results of explanatory binomial candidate model explaining which combination of factors will influence the ‘success‘.**
Additional file 4:
**Tables showing how Concentration of RNA (an equivalent of mRNA transferred into pre-amplification reaction) influences ‘success‘. A. Tested for all Genes and all Cycles together. B. Tested for each Gene independently and all Cycles together.**
Additional file 5:
**Figures showing how Copy number (copy number of cDNA used for pre-amplification) influences ‘success‘. A. Tested for all Genes and all Cycles together. B. Tested for each Gene independently and all Cycles together. C. Tested for all Genes and each Cycle independently.**
Additional file 6:
**Tables showing how number of Cycles (number of pre-amplification cycles) influences ‘success‘. A. Tested for all Genes and Concentrations together. B. Tested for each Gene independently.**
Additional file 7:
**A pivot table showing the success rate as a percentage for the possible combinations of Cycles and Concentrations for individual genes.** The additional information for Figure 1. Additional file 8:
**A standard curve of non-preamplified sample detected by 18S rRNA used in GE Dynamic Array 48.48.**

## Abbreviations

Cq: Cycle of quantification
EDTA: Ethylenediaminetetraacetic acid
gDNA: Genomic DNA
GeneNo: Gene number = different transcript
IFC: Integrated fluidic circuit
LOD: Limit of detection
Log_concentn: Log_2_ concentration of cDNA, presented as total RNA equivalent, used for pre-amplification
Log_copy: Log_2_ copy number of cDNA used for pre-amplification
LOQ: Limit of quantification
MB water: Molecular biology grade water
NTC: No template control
PCA: Principal component analysis
qPCR: Quantitative polymerase chain reaction
RIN: RNA integrity number
RT-: Negative control in reverse transcription, without reverse transcriptase
SOM: Kohonen self-organising map
SD: Standard deviation
STA: Specific target amplification
TE-LPA: Linear polyacrylamide carrier in TE buffer
