# Supplementary material for: Pre-amplification in the context of high-throughput qPCR gene expression experiment
Source: BMC Mol Biol. 2015 Mar 11;16:5. doi: 10.1186/s12867-015-0033-9 (PMC4365555; doi:10.1186/s12867-015-0033-9)
Supplement: Additional file 3: — Construction and results of explanatory binomial candidate model explaining which combination of factors will influence the ‘success‘. [file 12867_2015_33_MOESM3_ESM.pdf]

Additional File 3

## WHAT COMBINATION OF FACTORS WILL INFLUENCE SUCCESS?

An *explanatory* (using all possible terms) binomial candidate model was constructed:

Success ~ geneNo + donor + cycle + log_concentration + log_copy

An optimal model was then derived in SPSS using the backward stepwise method to eliminate 'non-significant' terms. Deriving an optimal model:

Success ~ geneNo + cycle + log_concentration

As donor and log_copy were eliminated, this is also a *predictive* model (all terms are known beforehand and controllable).

| Variables | B | S.E. | Wald | df | Sig. | Exp(B) | 95% C.I.for EXP(B) | |
|---|---|---|---|---|---|---|---|---|
| geneNo | | | 37.893 | 4 | 0.000 | | | |
| geneNo(1) | 0.358 | 0.38 | 0.891 | 1 | 0.345 | 1.431 | 0.68 | 3.012 |
| geneNo(2) | 2.111 | 0.441 | 22.953 | 1 | 0.000 | 8.257 | 3.481 | 19.584 |
| geneNo(3) | 1.708 | 0.418 | 16.704 | 1 | 0.000 | 5.518 | 2.432 | 12.516 |
| geneNo(4) | 1.803 | 0.423 | 18.197 | 1 | 0.000 | 6.067 | 2.65 | 13.892 |
| cycle | | | 64.653 | 3 | 0.000 | | | |
| cycle(15) | 2.672 | 0.42 | 40.513 | 1 | 0.000 | 14.464 | 6.353 | 32.929 |
| cycle(18) | 2.672 | 0.42 | 40.513 | 1 | 0.000 | 14.464 | 6.353 | 32.929 |
| cycle(21) | 0.543 | 0.316 | 2.942 | 1 | 0.086 | 1.721 | 0.925 | 3.199 |
| log_concn | 0.259 | 0.069 | 13.948 | 1 | 0.000 | 1.296 | 1.131 | 1.485 |
| Constant | -1.468 | 0.352 | 17.397 | 1 | 0.000 | 0.23 | | |

a Variable(s) entered on step 1: geneNo, donor, log_copy, cycle, log_concn.

**Classification Table(a)**

| | | Predicted | | |
|---|---|---|---|---|
| | | Success | | Percentage Correct |
| Observed | | Failure | Success | |
| Success | Failure | 62 | 57 | 52.1 |
| | Success | 30 | 251 | 89.3 |
| Overall Percentage | | 0.67 | 0.81 | 78.3 |

a The cut value is .500

The model has a sensitivity of 81% and a specificity of 67%.
