# Supplementary material for: Pre-amplification in the context of high-throughput qPCR gene expression experiment
Source: BMC Mol Biol. 2015 Mar 11;16:5. doi: 10.1186/s12867-015-0033-9 (PMC4365555; doi:10.1186/s12867-015-0033-9)
Supplement: Additional file 4: — Tables showing how Concentration of RNA (an equivalent of mRNA transferred into pre-amplification reaction) influences ‘success‘. A. Tested for all Genes and all Cycles together. B. Tested for each Gene independently and all Cycles together. [file 12867_2015_33_MOESM4_ESM.pdf]

Additional File 4

WILL CONCENTRATION OF RNA INFLUENCE SUCCESS?

A. *For all genes and all cycles together*

Concentration had a significant effect on the overall likelihood of success. Concentration was tested as a category (p=0.023), a variable (p=0.012), and a log transformed variable (p=0.001). In all cases was it significant for success. The categorical test results are displayed. The likelihood of success increases with the concentration.

concentration * Success Crosstabulation

			Success		Total
			Failure	Success	
concentn	.078	Count	32	48	80
		% within concentration	40.0%	60.0%	100.0%
	.320	Count	30	50	80
		% within concentration	37.5%	62.5%	100.0%
	1.250	Count	21	59	80
		% within concentration	26.3%	73.8%	100.0%
	5.000	Count	20	60	80
		% within concentration	25.0%	75.0%	100.0%
	20.000	Count	16	64	80
		% within concentration	20.0%	80.0%	100.0%
Total		Count	119	281	400
		% within concentration	29.8%	70.3%	100.0%

B. *For each gene independently and all cycles together*

Concentration had a significant effect on genes *RND1* ($p < 0.001$) and *CD83* ($p = 0.001$). Here increasing concentrations increased the likelihood of success. Gene *CD83* was still significant using the more conservative Fisher's Exact test.

concentration * Success * Gene Number Crosstabulation

Gene Number				Success	
				Failure	Success
RND1	concentration	.078	Count	12	4
			% within concentration	75.0%	25.0%
		.320	Count	10	6
			% within concentration	62.5%	37.5%
		1.250	Count	5	11
			% within concentration	31.3%	68.8%
		5.000	Count	7	9
			% within concentration	43.8%	56.3%
		20.000	Count	0	16
			% within concentration	0.0%	100.0%
	Total		Count	34	46
			% within concentration	42.5%	57.5%
CD83	concentration	.078	Count	8	8
			% within concentration	50.0%	50.0%
		.320	Count	2	14
			% within concentration	12.5%	87.5%
		1.250	Count	2	14
			% within concentration	12.5%	87.5%
		5.000	Count	1	15
			% within concentration	6.3%	93.8%
		20.000	Count	0	16
			% within concentration	0.0%	100.0%
	Total		Count	13	67
			% within concentration	16.3%	83.8%
