# Supplementary material for: Pre-amplification in the context of high-throughput qPCR gene expression experiment
Source: BMC Mol Biol. 2015 Mar 11;16:5. doi: 10.1186/s12867-015-0033-9 (PMC4365555; doi:10.1186/s12867-015-0033-9)

Additional File 5

WILL COPY NUMBER INFLUENCE SUCCESS?

A. *For all genes and all cycles together*

Copy number (log of copy number) did not have a significant effect on the overall likelihood of success (p = 0.3218).

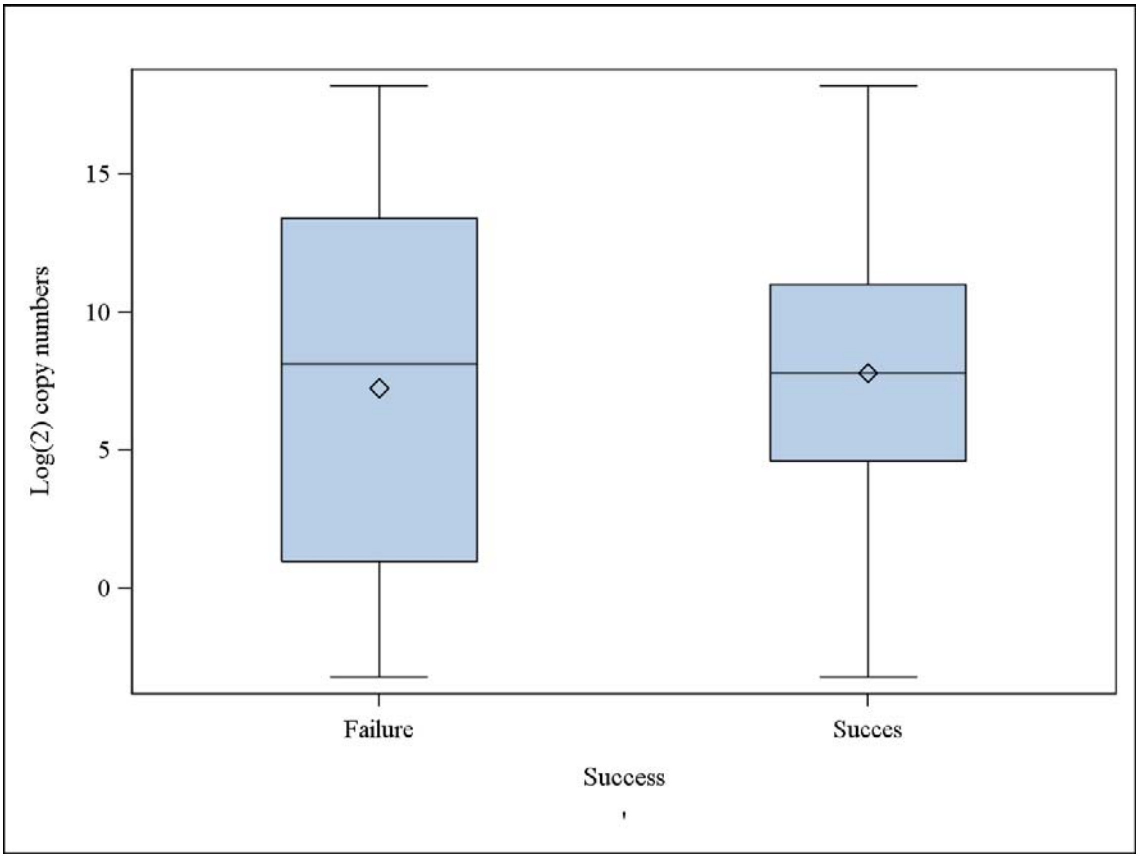

B. For each gene independently and all cycles together

Copy number only had a significant effect on genes *RND1* ($p = 0.0001$) and *CD83* ($p = 0.0004$). Here increased copy number increased the likelihood of success.

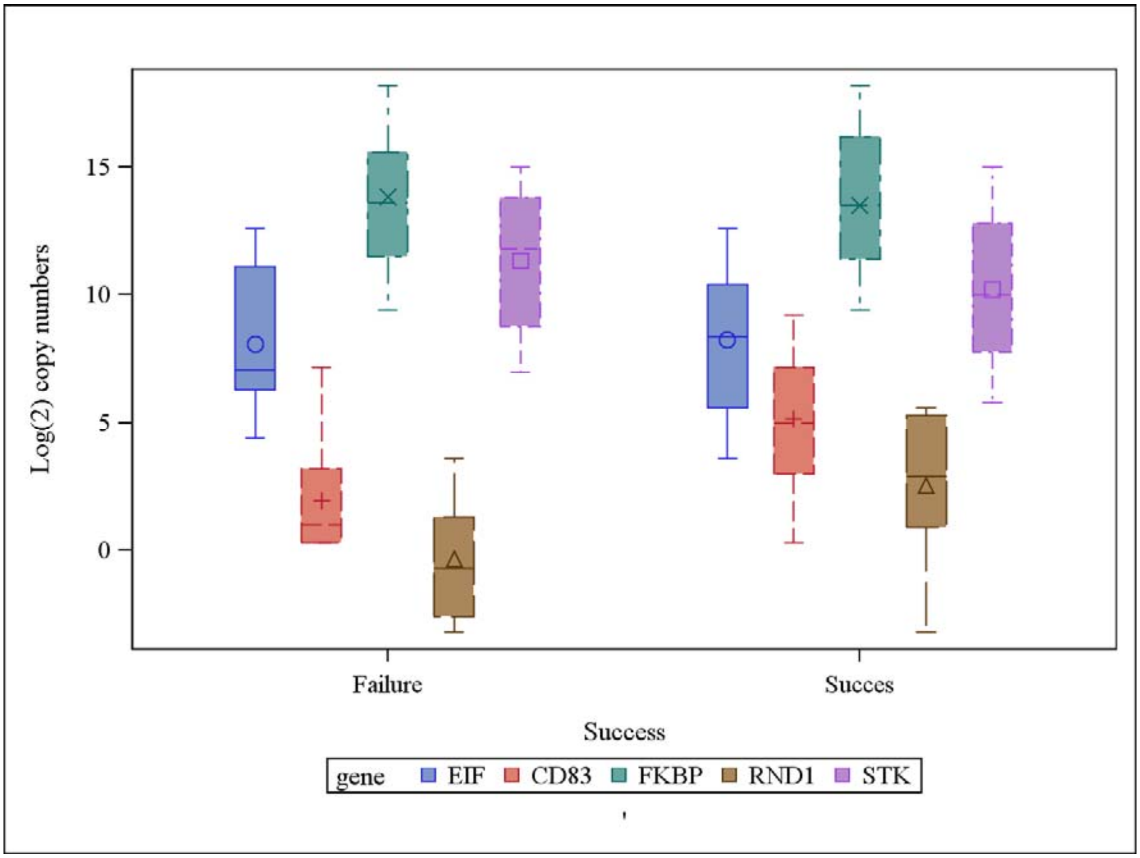

C. For all genes and each cycle independently

Copy number (log copy number) was significant for cycles 15 (p = 0.0006), 18 (p = 0.0002), and 24 (p = 0.0007). Whereas the likelihood of success increases with increasing copy number for cycles 15 and 18, it decreases for cycle 24. The contradictory directions for individual cycles means there is no overall significant effect above. Rings are outliers.

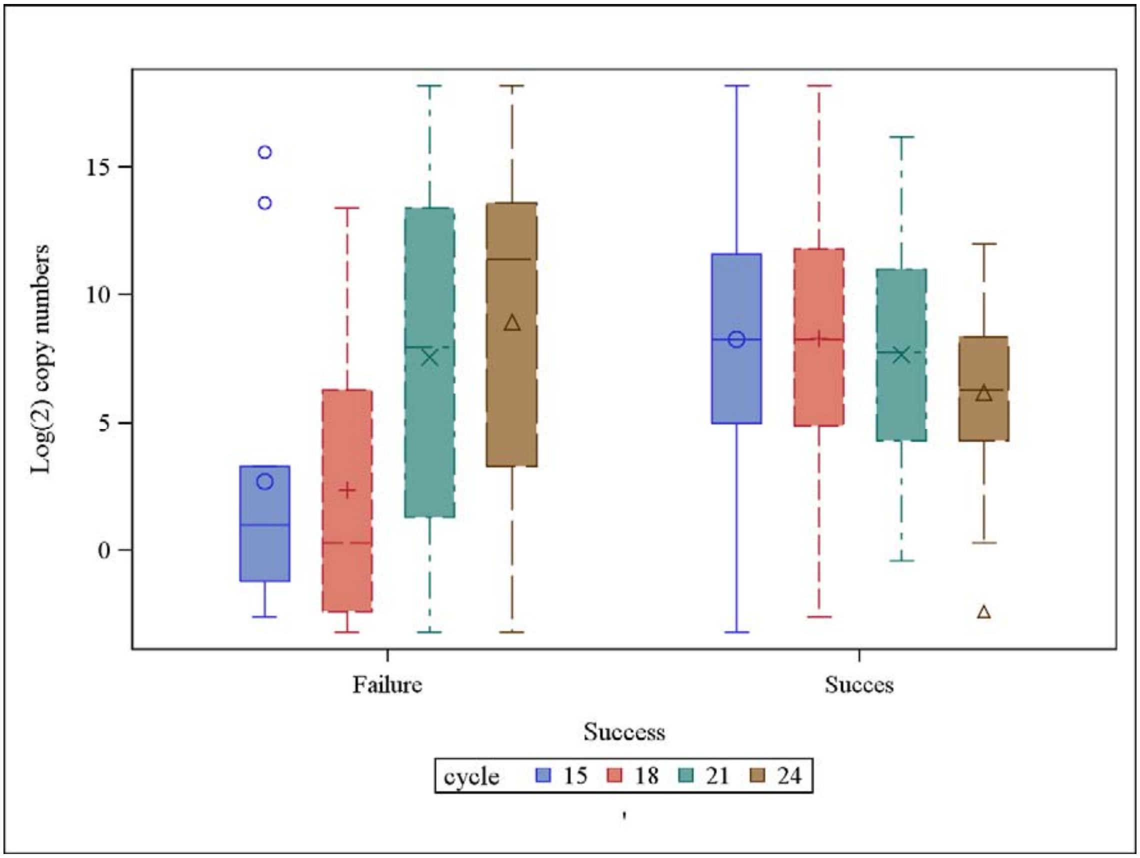

Supplement: Additional file 5: — Figures showing how Copy number (copy number of cDNA used for pre-amplification) influences ‘success‘. A. Tested for all Genes and all Cycles together. B. Tested for each Gene independently and all Cycles together. C. Tested for all Genes and each Cycle independently. [file 12867_2015_33_MOESM5_ESM.pdf]
