# Supplementary material for: Pre-amplification in the context of high-throughput qPCR gene expression experiment
Source: BMC Mol Biol. 2015 Mar 11;16:5. doi: 10.1186/s12867-015-0033-9 (PMC4365555; doi:10.1186/s12867-015-0033-9)
Supplement: Additional file 6: — Tables showing how number of Cycles (number of pre-amplification cycles) influences ‘success‘. A. Tested for all Genes and Concentrations together. B. Tested for each Gene independently. [file 12867_2015_33_MOESM6_ESM.pdf]

Additional File 6

WILL NUMBER OF CYCLES INFLUENCE SUCCESS?

A. *For all genes and concentrations together*

The number of cycles had a highly significant effect on the overall likelihood of success ($p < 0.001$). Increasing cycle numbers decreased the likelihood of success.

cycle * Success Crosstabulation

			Success		Total
			Failure	Success	
cycle	15	Count	11	89	100
		% within cycle	11.0%	89.0%	100.0%
	18	Count	11	89	100
		% within cycle	11.0%	89.0%	100.0%
	21	Count	43	57	100
		% within cycle	43.0%	57.0%	100.0%
	24	Count	54	46	100
		% within cycle	54.0%	46.0%	100.0%
Total		Count	119	281	400
		% within cycle	29.8%	70.3%	100.0%

B. *For each genes independently*.

Cycle number had a significant effect on genes *EIF3M* ($p = 0.001$), *STK10* ($p < 0.001$), and *FKBP* ($p < 0.001$). Increasing cycle numbers drastically decreased the likelihood of success (as above)**.**

cycle * Success * Gene Number Crosstabulation

Gene Number				Success		Total
				Failure	Success	
EIF3M	cycle	15	Count	0	20	20
			% within cycle	0.0%	100.0%	100.0%
		18	Count	1	19	20
			% within cycle	5.0%	95.0%	100.0%
		21	Count	7	13	20
			% within cycle	35.0%	65.0%	100.0%
		24	Count	9	11	20
			% within cycle	45.0%	55.0%	100.0%
	Total		Count	17	63	80
			% within cycle	21.3%	78.8%	100.0%
STK10	cycle	15	Count	0	20	20
			% within cycle	0.0%	100.0%	100.0%
		18	Count	0	20	20
			% within cycle	0.0%	100.0%	100.0%
		21	Count	6	14	20
			% within cycle	30.0%	70.0%	100.0%
		24	Count	10	10	20
			% within cycle	50.0%	50.0%	100.0%
	Total		Count	16	64	80
			% within cycle	20.0%	80.0%	100.0%
FKBP	cycle	15	Count	2	18	20
			% within cycle	10.0%	90.0%	100.0%
		18	Count	2	18	20
			% within cycle	10.0%	90.0%	100.0%
		21	Count	16	4	20
			% within cycle	80.0%	20.0%	100.0%
		24	Count	19	1	20
			% within cycle	95.0%	5.0%	100.0%
	Total		Count	39	41	80
			% within cycle	48.8%	51.3%	100.0%
