# Supplementary material for: Pre-amplification in the context of high-throughput qPCR gene expression experiment
Source: BMC Mol Biol. 2015 Mar 11;16:5. doi: 10.1186/s12867-015-0033-9 (PMC4365555; doi:10.1186/s12867-015-0033-9)
Supplement: Additional file 7: — A pivot table showing the success rate as a percentage for the possible combinations of Cycles and Concentrations for individual genes. The additional information for Figure 1. [file 12867_2015_33_MOESM7_ESM.pdf]

Additional File 7

DETAILED TABLE FOR FIGURE 1

A pivot table showing the success rate as a percentage for the possible combinations of Cycles and Concentrations for individual genes. The additional information for Figure 1.

**Proportion of success**	**Concentration**					
**Cycle**	**0.078**	**0.32**	**1.25**	**5**	**20**	**Grand Total**
**RND1 S**						
**15**	0.50	0.50	0.75	0.75	1.00	0.70
**18**	0.25	0.50	0.75	0.75	1.00	0.65
**21**	0.00	0.25	0.75	0.50	1.00	0.50
**24**	0.25	0.25	0.50	0.25	1.00	0.45
**CD83 S**						
**15**	0.25	1.00	1.00	1.00	1.00	0.85
**18**	0.75	1.00	1.00	1.00	1.00	0.95
**21**	0.50	0.75	0.75	1.00	1.00	0.80
**24**	0.50	0.75	0.75	0.75	1.00	0.75
**EIF3M S**						
**15**	1.00	1.00	1.00	1.00	1.00	1.00
**18**	1.00	0.75	1.00	1.00	1.00	0.95
**21**	0.75	0.00	0.50	1.00	1.00	0.65
**24**	0.50	0.75	0.75	0.75	0.00	0.55
**STK10 S**						
**15**	1.00	1.00	1.00	1.00	1.00	1.00
**18**	1.00	1.00	1.00	1.00	1.00	1.00
**21**	0.50	0.25	0.75	1.00	1.00	0.70
**24**	0.75	0.75	0.75	0.25	0.00	0.50
**FKBP S**						
**15**	1.00	1.00	0.75	0.75	1.00	0.90
**18**	1.00	0.75	0.75	1.00	1.00	0.90
**21**	0.25	0.25	0.25	0.25	0.00	0.20
**24**	0.25	0.00	0.00	0.00	0.00	0.05
**Grand Total**	0.60	0.63	0.74	0.75	0.80	0.70
